# Quantitative flow chamber system for evaluating in vitro biofilms and the kinetics of *S. aureus* biofilm formation in human plasma media

**DOI:** 10.1186/s12866-021-02379-9

**Published:** 2021-11-11

**Authors:** Werasak Sutipornpalangkul, Kohei Nishitani, Edward M. Schwarz

**Affiliations:** 1 The Center for Musculoskeletal Research, University of Rochester, Rochester, NY USA; 2 Department of Orthopaedic Surgery, Faculty of Medicine, Siriraj Hospital, Mahidol University, Bangkok, Thailand; 3 Department of Orthopaedic Surgery, Graduate School of Medicine, Kyoto University, Kyoto, Japan

**Keywords:** In vitro biofilm, *Staphylococcus aureus*, Scanning Electron microscopy, Bioluminescence

## Abstract

**Background:**

It has been well established that biofilm formation on orthopaedic implants is a critical event in the pathogenesis of orthopaedic infections, yet the natural history of this process with respect to bacterial adhesion, proliferation, and glycocalyx matrix production remains poorly understood. Moreover, there are no quantitative methods yet available to assess the differences in biofilm formation between different bacterial strains or implant materials. Consequently, this study aimed to investigate the natural history of *S. aureus* in in vitro biofilm formation in human plasma media using a flow chamber system. Bioluminescent *S. aureus* strains were used to better understand the bacterial growth and biofilm formation on orthopaedic materials. Also, the effects of human plasma media were assessed by loading the chamber with Tryptic Soy Broth with 10% human plasma (TSB + HP).

**Results:**

Scanning electron microscopy (SEM) was utilized to assess the morphological appearance of the biofilms, revealing that *S. aureus* inoculation was required for biofilm formation, and that the phenotypes of biofilm production after 24 h inoculation with three tested strains (SH1000, UAMS-1, and USA300) were markedly different depending on the culture medium. Time course study of the bioluminescence intensity (BLI) and biofilm production on the implants due to the UAMS-1 and USA300 strains revealed different characteristics, whereby UAMS-1 showed increasing BLI and biofilm growth until peaking at 9 h, while USA300 showed a rapid increase in BLI and biofilm formation at 6 h. The kinetics of biofilm formation for both UAMS-1 and USA300 were also supported and confirmed by qRT-PCR analysis of the 16S rRNA gene. Biofilms grown in our flow chamber in the plasma media were also demonstrated to involve an upregulation of the biofilm-forming-related genes *icaA, fnbA,* and *alt*. The BLI and SEM results from K-wire experiments revealed that the in vitro growth and biofilm formation by UAMS-1 and USA300 on stainless-steel and titanium surfaces were virtually identical.

**Conclusion:**

We demonstrated a novel in vitro model for *S. aureus* biofilm formation with quantitative BLI and SEM outcome measures, and then used this model to demonstrate the presence of strain-specific phenotypes and its potential use to evaluate anti-microbial surfaces.

## Background

Infection remains a major complication of orthopaedic surgery, with ~ 50–60% of infections caused by *Staphylococcus aureus* [1–3]. Especially for post-arthroplasty, infections occur in 1–5% of cases, either from primary arthroplasty or revision arthroplasty [4, 5]. Most of these infections are caused by *S. aureus* [6, 7]. The treatment of post-arthroplasty infection typically requires extensive medical and surgical care, and involves significant healthcare costs, prolonged disability/rehabilitation, and significantly worse outcomes [8]. It is known that bacteria can adhere to orthopaedic implants, whereupon they form biofilms that prevent the penetration of immune cells and antibiotics [9, 10]. Although it has been well established that biofilm formation on orthopaedic implants is a critical event in the pathogenesis of orthopaedic infections [11], the natural history of this process with respect to bacterial adhesion, proliferation, and glycocalyx matrix production remains poorly understood [12–14]. Moreover, there are no quantitative methods yet available to assess the differences in biofilm formation between different bacterial strains or implant materials. While there is extensive literature on this topic based on the results from static in vitro biofilm assays [15–20], a previous study involving scanning electron microscopy (SEM) evaluation of samples revealed that some regions that were quantified as positive in crystal-violet staining did not contain any biofilm, and most strains did not produce glycocalyx in static cultures [21]. Thus, we chose to investigate the natural history of *S. aureus* dynamics in in vitro biofilm formation using a flow chamber system and quantitative analysis by SEM. After confirming glycocalyx production in the flow chamber, we aimed to characterize the kinetics and the phenotypes of biofilm formation by three different *S. aureus* strains, namely SH1000, UAMS-1, USA300. We used two bioluminescent *S. aureus* strains, namely Xen40 and USA300 LAC::lux, to better understand the nature of this biomarker of bacterial growth and biofilm formation. Bioluminescence imaging (BLI), assessment of the morphological appearance by SEM, and quantification of the bacteria load by qRT-PCR were the analytical techniques we used to study the kinetics and phenotypes of the *S. aureus* biofilms. Finally, we compared the natural history of biofilm formation on different Kirschner (K) wires to test the hypothesis that titanium (Ti) [21] is more resistant to *S. aureus* colonization than stainless steel (SS).

## Results

### Robust biofilm formation in human plasma media

**Fig. 1:**
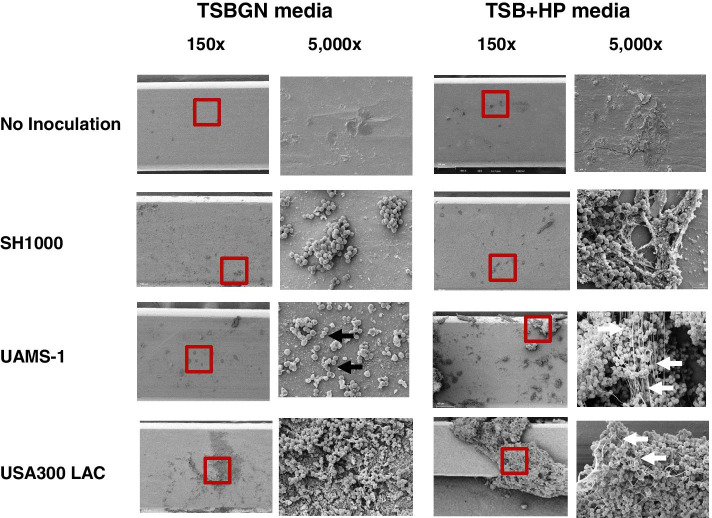
Glucose/salt versus plasma requirements for in vitro biofilm formation on stainless steel by different *S. aureus* strains assessed by SEM at 24 h. Stainless-steel pins were placed in the flow chamber containing TSBGN or TSB + HP media without bacteria, or inoculated with SH1000, UAMS-1, or USA300 *S. aureus*, and incubated for 24 h at a flow rate of 0.2 ml/min at 37 °C. Afterwards, the pins were harvested and processed for SEM. Representative SEM images of two independent experiments are shown with the region of interest (ROI; red box in the photo) of the biofilm in the left panels obtained at 150x, and at 5000x in the right panels. Of note is that SH1000 produced a greater biofilm in TSBGN that was phenotypically similar to that produced in TSB + HP. In contrast, UAMS-1 failed to produce a biofilm in TSBGN, as evidenced by the sparse cell clusters (black arrows), but generated a robust biofilm containing fibres (white arrows) in TSB + HP. USA300 produced robust biofilms in both media, but fibres (white arrows) were only present when grown in TSB + HP.

**Fig. 2:**
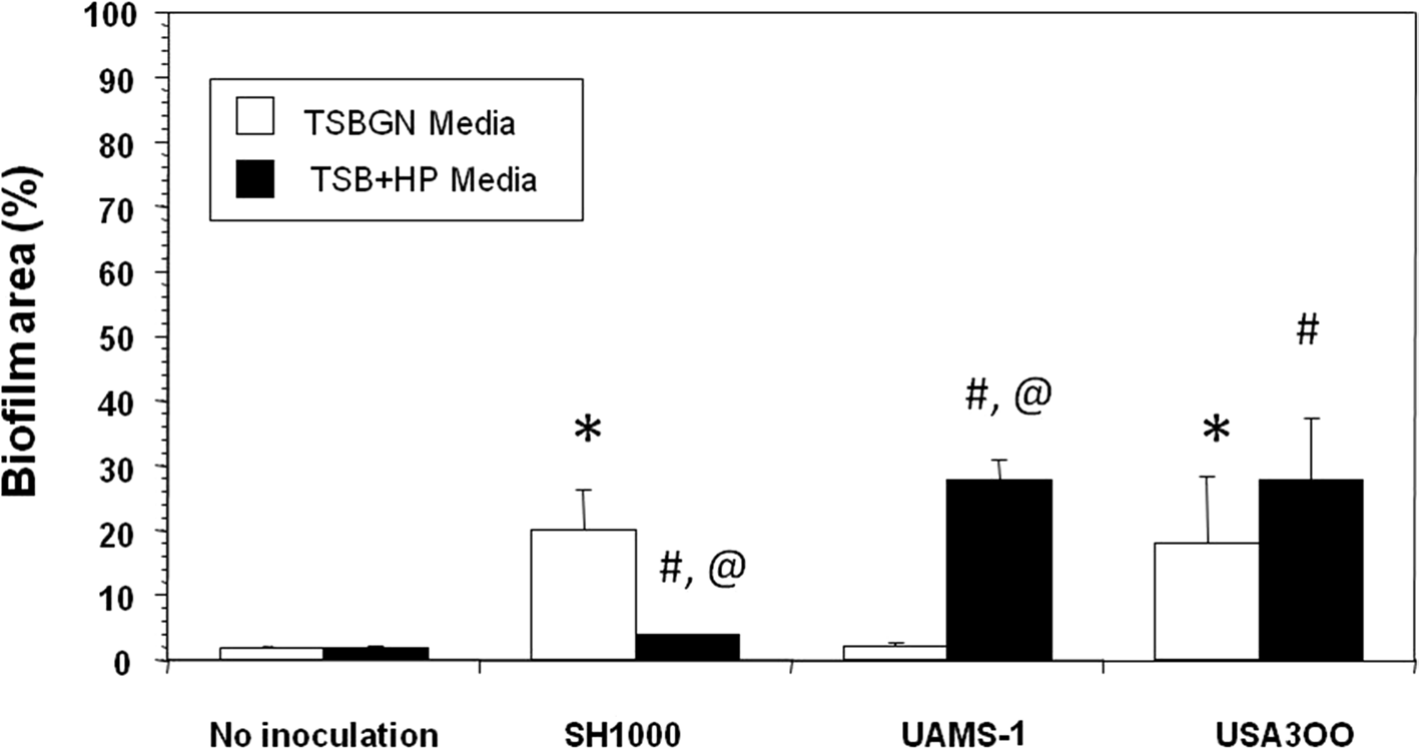
Quantification of the in vitro biofilm formation on stainless-steel pins. A 0.25 mm^2^ ROI in the 150x SEM images of the pins (*n* = 4) described in Fig. 1 was used to quantify the % surfaced covered by the biofilm using NIH Image, and the data are presented as the mean +/− SD (*p < 0.05 vs. no inoculation in TSBGN media; ^#^*p* < 0.05 vs. no inoculation in TSB + HP media; ^@^ *p* < 0.05 TSBGN vs. TSB + HP for each strain)

Assessment of the SEM images revealed that *S. aureus* inoculation was required for biofilm formation, and that the phenotypes of biofilm production after 24 h inoculation by the three tested strains were markedly different depending on the culture media (Fig. 1). In the absence of inoculated *S. aureus* bacteria, there was no biofilm on the implant pins, both in tryptic soy broth with 0.5% dextrose and 3% NaCl (TSBGN) and tryptic soy broth with 10% human plasma (TSB + HP) media. SH1000 produced more biofilm in TSBGN, and its biofilm in TSB + HP did not contain a network of fibres. UAMS-1 biofilm formation in TSBGN was scant and limited to small clusters of bacteria that did not contain a matrix, while its biofilm was robust in TSB + HP and extensively covered by a matrix containing networks of fibres. USA300 produced robust biofilms in both culture media; however, networks of fibres were only produced in the TSB + HP media. SEM quantification using NIH Image software confirmed these significant differences (Fig. 2). In the TSBGN media, SH1000 and USA300 formed robust biofilms with statistically significant differences compared to the non-inoculated bacteria and UAMS-1. In the TSB + HP media, all three strains (SH1000, UAMS-1, and USA300) formed more biofilm than in the case with the non-inoculated bacteria with statistical significance. When comparing each strain in these two different media, SH1000 had less biofilm formation in TSB + HP media compared to in TSBGN media. Both UAMS-1 and USA300 had robust biofilm formation in TSB + HP media. UAMS-1 produced biofilm statistically differently in TBS + HP media compared to in TSBGN media. However, USA300 showed no significance difference in biofilm formation in TSB + HP media compared to in TSBGN media.

### Fibronectin networks formed in the biofilm matrix

**Fig. 3:**
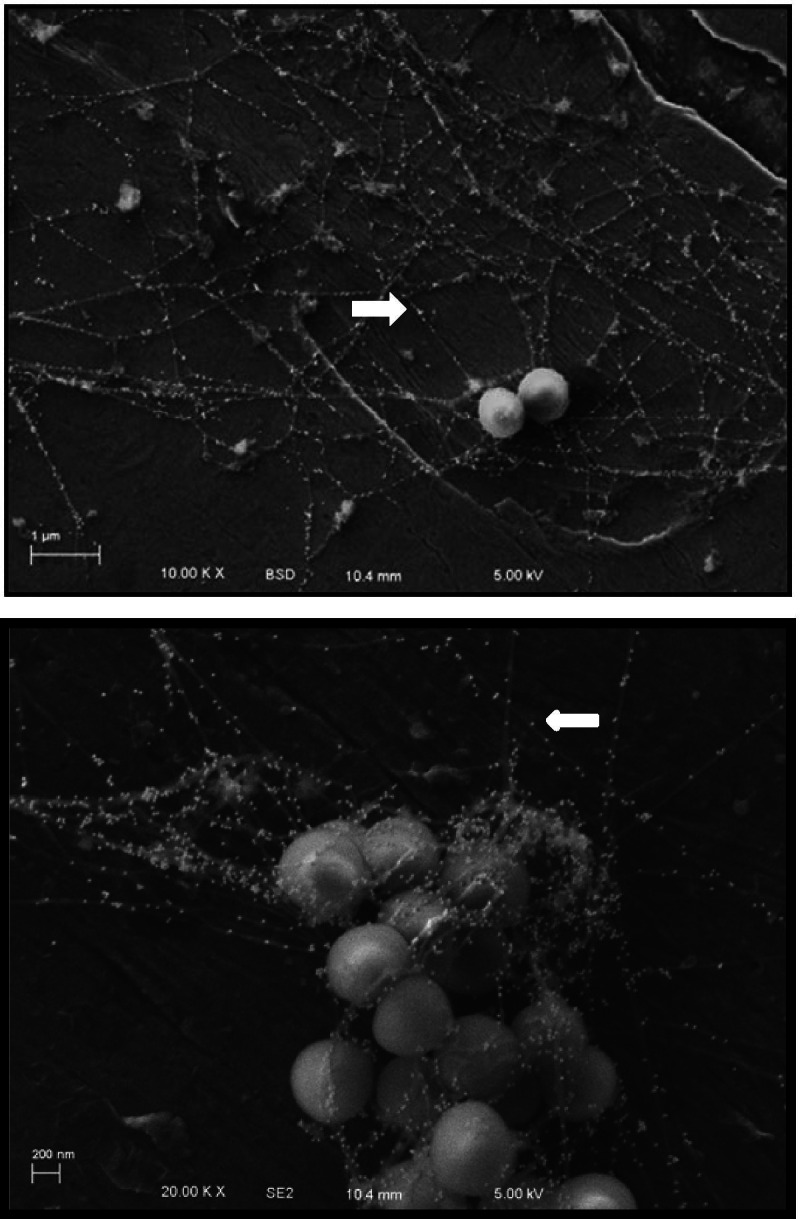
Immunogold-labelling for fibrinogens from the in vitro biofilm formation on stainless-steel pins. Stainless-steel pins were placed in the flow chamber containing TSB + HP media and inoculated with UAMS-1 *∆spa S. aureus*, and incubated for 9 h at a flow rate of 0.2 ml/min at 37 °C. Afterwards, the pins were harvested and processed for immunogold-labelling. Representative SEM images of two independent experiments are shown. The biofilm in the upper panels were obtained at 10,000x, and at 20,000x in the lower panels. The network fibres in the matrix of the biofilm were coated by gold particles (white arrows), which directly attach to specific antibodies of the fibrinogen molecules

To identify the network components in the biofilm, we performed in vitro biofilm formation analysis using the UAMS-1 *∆spa* strain, which decreased the non-specific binding of 1:100 fibrinogen tagged with goat anti-rabbit 30 nm gold secondary. Figure 3 shows that all the networks were coated by a bead of gold particles. Therefore, the structures of our biofilms were confirmed as comprising fibrinogen networks.

### Time course of in vitro biofilm formation

**Fig. 4:**
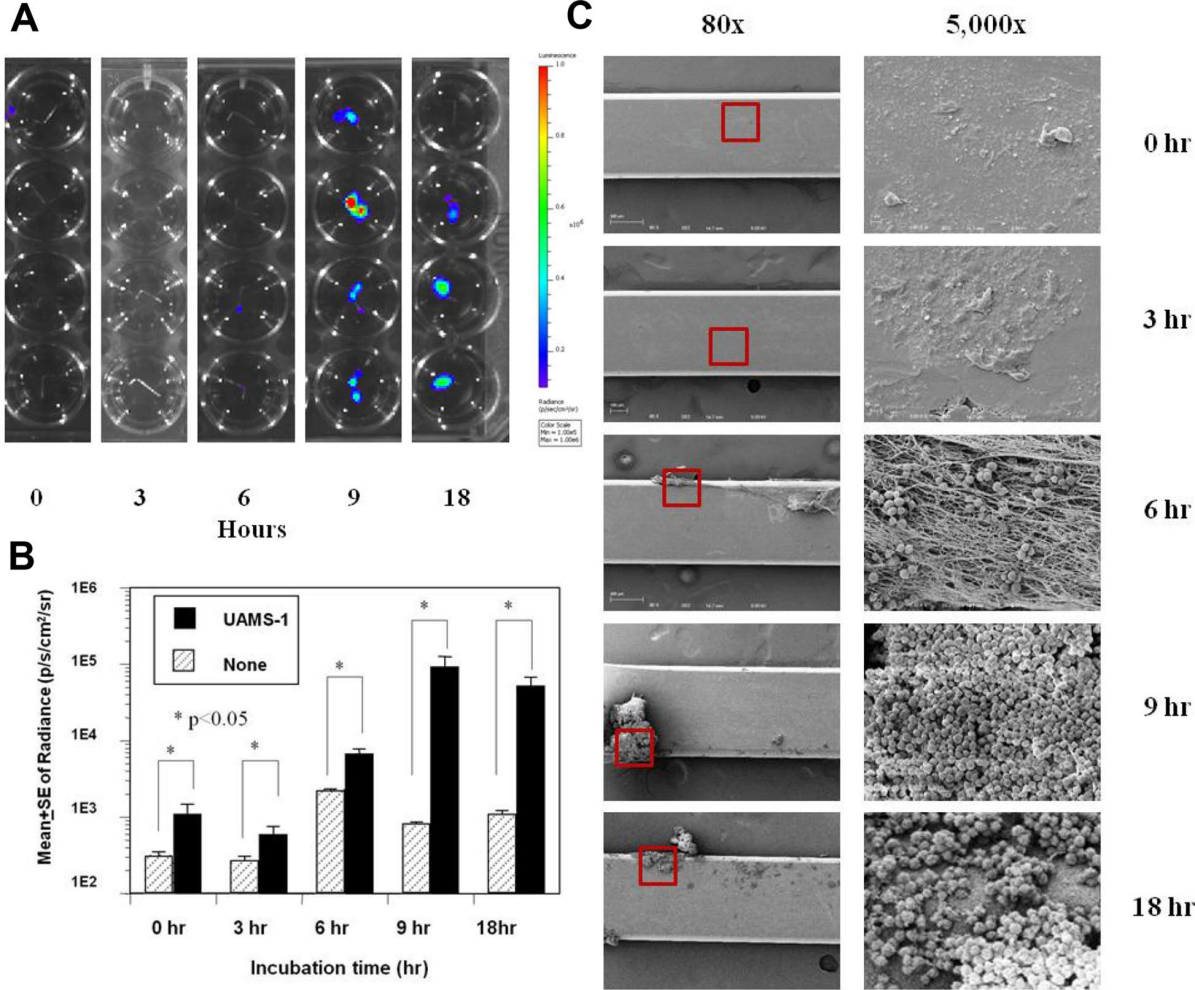
Kinetics of UAMS-1 in the in vitro biofilm formation on stainless steel. Bioluminescent UAMS-1 (Xen40) was inoculated (1 × 10^6^ cfu/ml) in the flow chamber containing TSB + HP and stainless-steel pins, which were harvested at the indicated time (*n* = 4). **A** Heat map images of the harvested pins in a microtiter plate during bioluminescent imaging (BLI exposure time 60 s, colour scale 1e5–1e6). **B** Quantification of the BLI signal (p/s/cm^2^/sr logarithmic scale) in the 1 cm^2^ ROI is presented as the mean +/− SEM (*p < 0.05). **C** Representative SEM images of the pin are shown at 80x (left), and the ROIs containing biofilm (red box) are shown in the SEM at 5000x (right). Note that a significant biofilm in the form of a precipitated matrix was formed immediately (0 h), while bacterial adherence to this matrix did not occur until 6 h, and biofilm formation peaked at 9 h

**Fig. 5:**
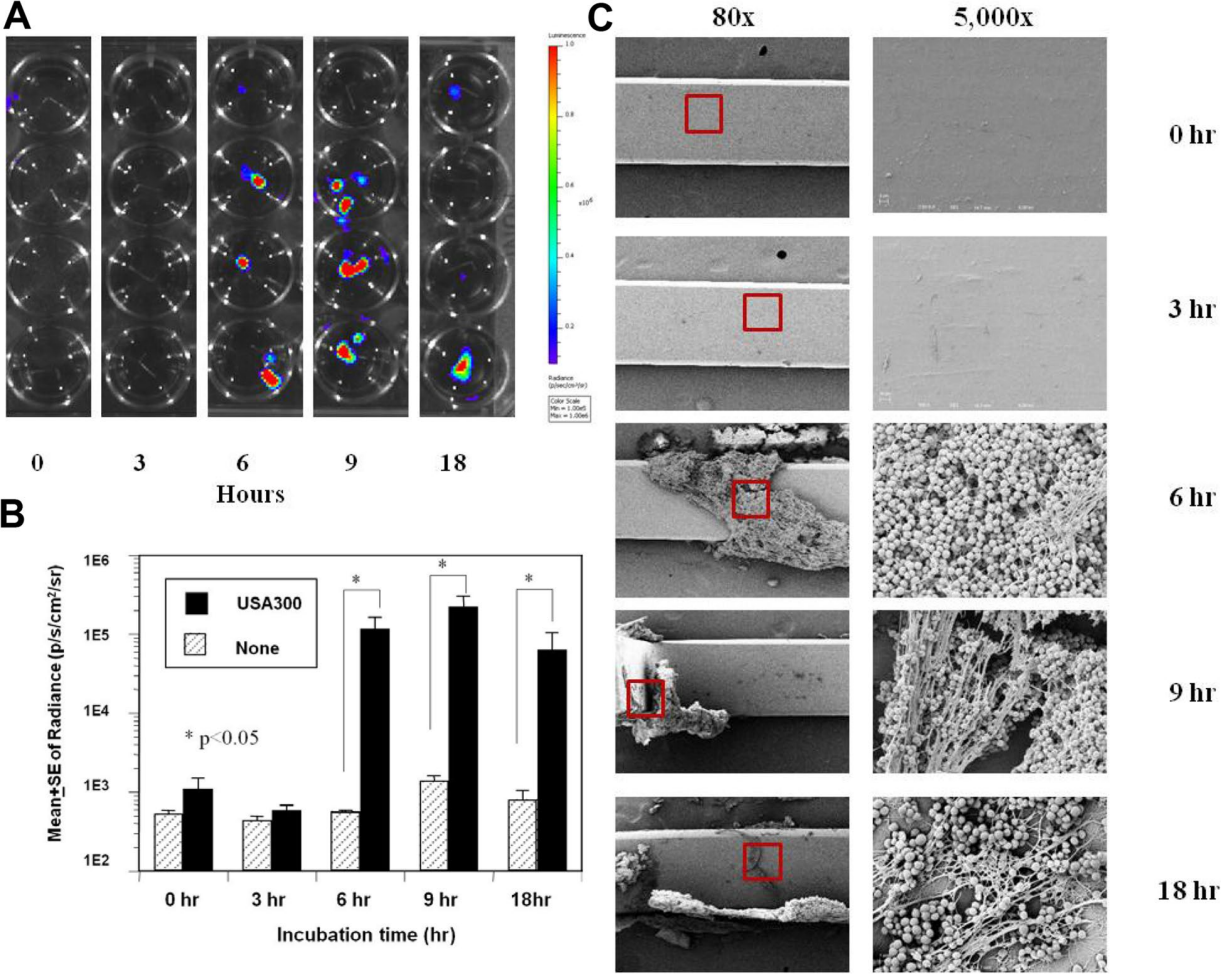
Kinetics of USA300 in the in vitro biofilm formation on stainless steel. Bioluminescent USA300 (USA300 LAC:lux) was inoculated in the flow chamber as described in Fig. 3, and the stainless-steel pins were harvested at the indicated time (*n* = 4). **A** Heat map images of the harvested pins in a microtiter plate during bioluminescent imaging (BLI exposure time 60 s, colour scale 1e5–1e6). **B** Quantification of the BLI signal (p/s/cm^2^/sr logarithmic scale) in the 1 cm^2^ ROI is presented as the mean +/− SEM (*p < 0.05). **C** Representative SEM images of the pin are shown at 80x (left), and the ROIs containing biofilm (red box) are shown in the SEM at 5000x (right). Note that significant biofilm formation did not occur until the bacteria had adhered to the pin at 6 h. While fibril networks formed on top of the bacteria thereafter, this did not markedly increase the BLI signal from its peak at 6 h

**Fig. 6:**
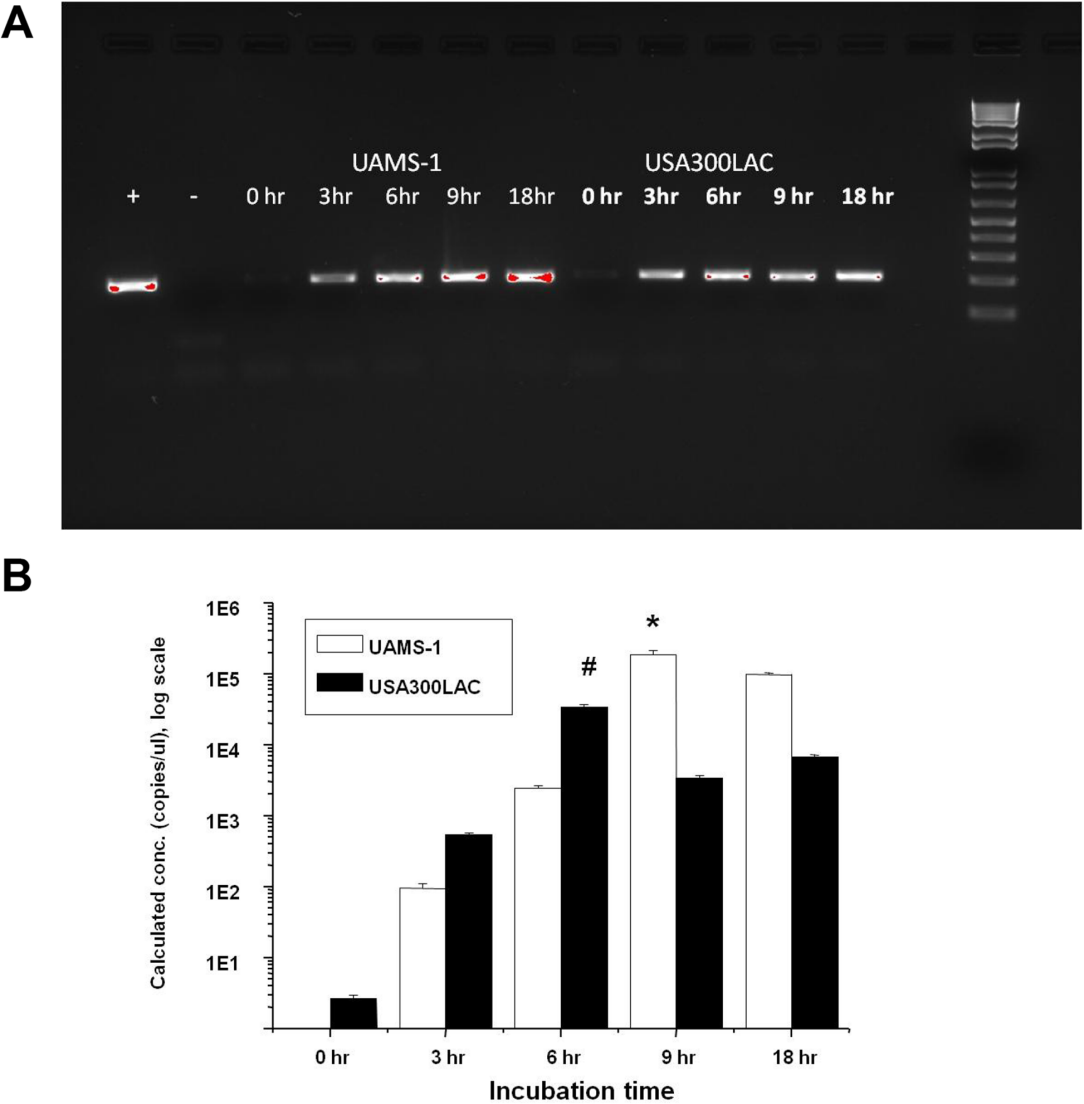
Kinetics of 16S rRNA for UAMS-1 and USA300 in the in vitro biofilm formation on stainless steel. Bioluminescent UAMS-1 and USA300 (USA300 LAC:lux) were inoculated in the flow chamber as described in Figs. 3 and 4, and the stainless-steel pins were harvested at the indicated time (*n* = 4). RNA was extracted and after that cDNA was generated by iScript kits. Electrophoresis and quantitative real-time PCR were done using 16S rRNA primers. **A** Images from the agarose gel electrophoresis are shown for UAMS-1 (left-side), and USA300 (right side). **B** Graph of the quantification real-time PCR analyses of UAMS-1 and USA300. Note that a significant amount of 16S rRNA occurred at 3 h and then increased in a time-dependent manner for both strains. UAMS-1 had its peak at 9 h, while USA300 peaked at 6 h (*p < 0.05 vs. other inoculation times in UAMS-1; ^#^*p* < 0.001 vs. other inoculation times in USA300)

Time course study of the BLI and biofilm production by UAMS-1 and USA300 revealed that UAMS-1 adhered to the implant immediately via secondary attachment to the fibronectin fibres that were formed directly on the pin, with an increasing BLI and biofilm growth until peaking at 9 h (Fig. 4A–C). In contrast, biofilm formation by USA300 commenced via direct bacterial adherence on to the pin after 3 h, and showed a rapid increase in BLI and biofilm formation at 6 h, together with a similar peak at 9 h to that observed with UAMS-1 (Fig. 5A–C). These kinetics of biofilm formation were further confirmed by measurement of the RNA of the living bacteria in the biofilm on the pins (Fig. 6). Figure 6A and B present the gel electrophoresis results of the PCR products and a graph showing the results from the quantification real-time PCR analysis of 16S rRNA from the UAMS-1 and USA300 biofilms, respectively. The amount of UAMS-1 16S rRNA on the pins increased according to the incubation time, peaking at 9 h, while USA300 showed a significantly higher amount of 16S rRNA at 6 h.

### Biofilm-related genes expression as assessed by qRT-PCR analysis

**Fig. 7:**
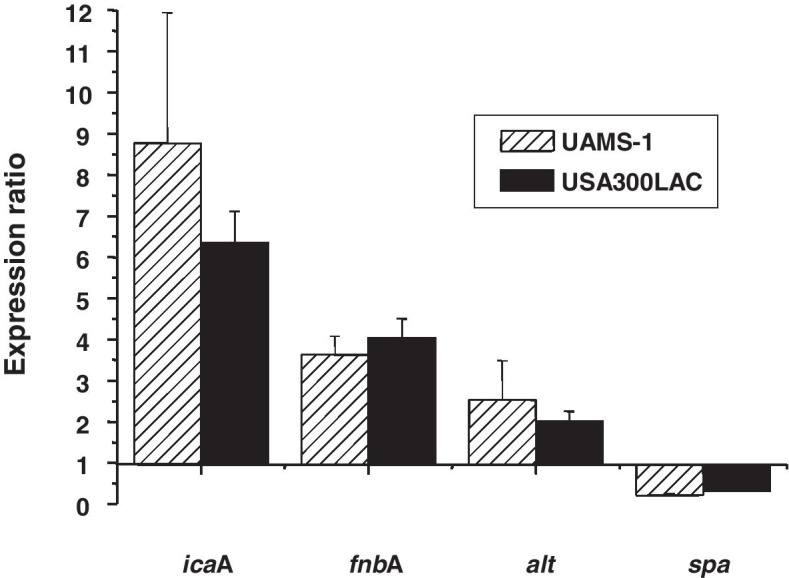
Relative expression levels of UAMS-1 and USA300 genes in the in vitro biofilm as determined by real-time PCR. Expression levels of the *icaA, fnbA*, *alt*, and *spa* genes were determined by real-time PCR. The relative expression levels are illustrated as the ratio of the expression level observed in the biofilm versus in the stationary-phase planktonic culture. The relative expression levels were calculated by comparison of the level of *gyrB* expression in the same cDNA preparations

The relative expression of the biofilm-related genes *icaA, fnbA*, *spa*, and *alt* are shown in Fig. 7. These comparisons were achieved using RNA isolated from planktonic bacteria and from biofilms on the pins. Most of the biofilm-related genes (*icaA, fnbA*, and *alt*) showed a high expression in the biofilms from both UAMS-1 and USA300 compared to with the planktonic bacteria. However, in our flow chamber, we found that the *spa* gene was downregulated in the biofilms compared to with the planktonic bacteria.

### Biofilms on different orthopaedic materials

**Fig. 8:**
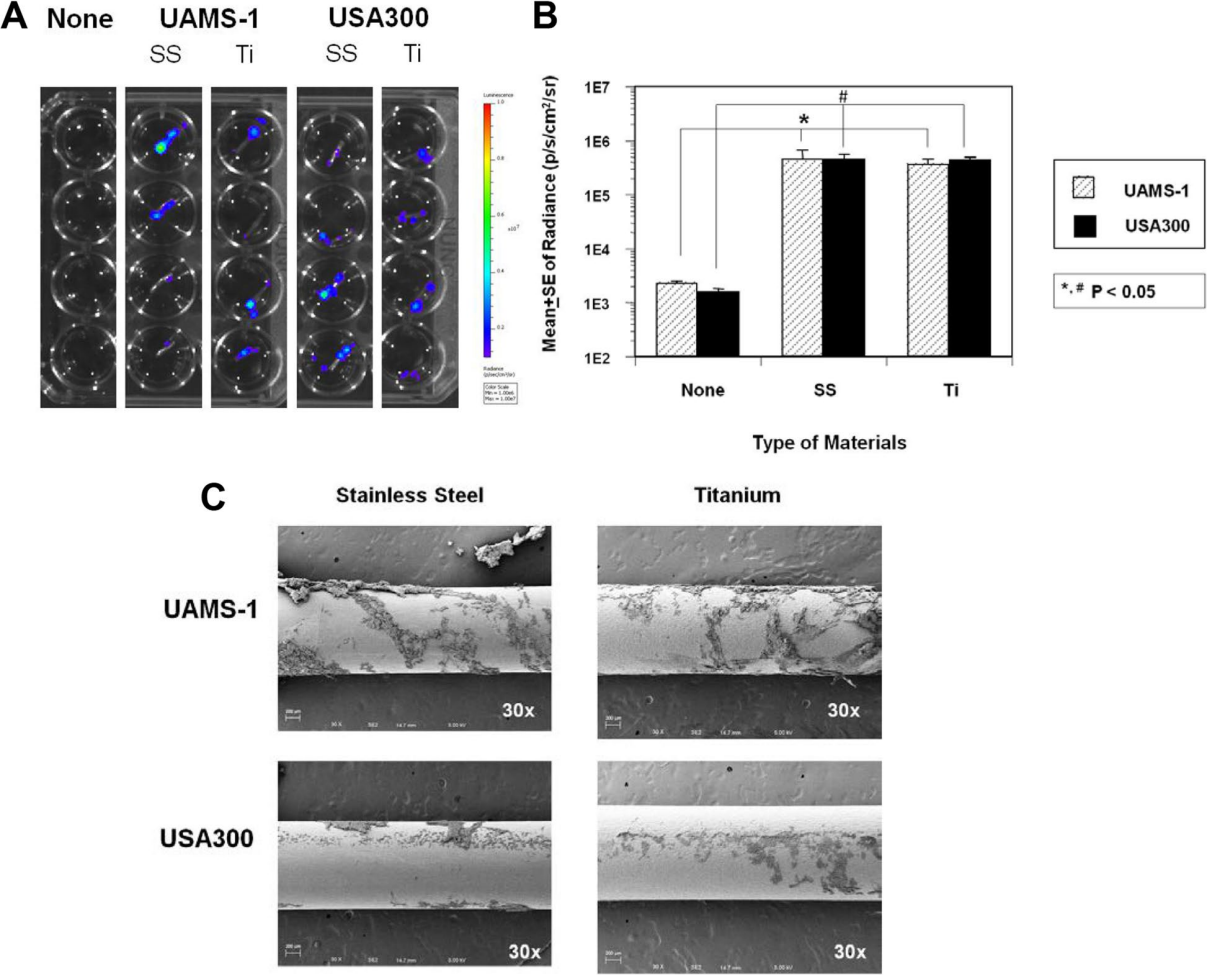
UAMS-1 and USA300 produced similar biofilms on stainless-steel (SS) and titanium (Ti) K-wires. Bioluminescent UAMS-1 or USA300 were inoculated in the flow chamber containing K-wires made of SS or Ti as described in Fig. 3. **A** Heat map images of the harvested K-wires in a microtiter plate during bioluminescent imaging (BLI exposure time 60 s, colour scale 1e6–1e7). **B** Quantification of the BLI signal (p/s/cm2/sr logarithmic scale) in the 1 cm^2^ ROI is presented as the mean +/− S.E. (*p < 0.05). **C** Representative SEM images of the biofilm on the implant are shown at 30x. No remarkable differences between the biofilm formed on SS vs. Ti were observed

Titanium (Ti) is commonly used in orthopaedic surgery as it is considered more resistant to biofilm formation than stainless steel (SS). However, the BLI and SEM results from the K-wire experiments revealed that the in vitro growth and biofilm formation by UAMS-1 and USA300 on SS and Ti surfaces were virtually identical (Fig. 8).

## Discussion

Based on the in vitro biofilm assay system, the best biofilm assay system should be representative of what occurs in vivo. In our previous studies, a flow chamber with a continuous flow of media was proven to be a better system to grow dynamic biofilms on orthopaedic materials [22]. Our findings in this study revealed that in the presence of human plasma in the media, *S. aureus* could dramatically form biofilms on implants. This result is line with the findings in a previous report by Chen et al. [13], who reported that with human plasma-containing media, *S. aureus* formed reproducible and robust biofilms, both in flow chambers and in static well plates. To the best of our knowledge, this is the first study to demonstrate that a matrix of these biofilms composed of networks of fibres is formed by the bacteria and then adheres on to the pins. These fibre networks were identified as fibronectin networks by immunogold-labelling. Also, 9 h incubation was found to be the most appropriate time for biofilm formation in this study based on evidence from the BLI, SEM, and qRT-PCR analyses. Both UAMS-1 and USA300 could form biofilms similarly on SS and Ti orthopaedic materials.

From our findings, the mechanisms by which human plasma promotes the dramatic growth of *S. aureus* biofilms might be explained by the presence of fibrinogen, which is one of the major components in human plasma. We suggest that the planktonic growth of *S. aureus* in our flow chamber might have led to the upregulation of surface adhesions, especially fibrinogen adherence factors. Therefore, clumps of *S. aureus* occurred within the fibrinogen networks and attached to our orthopaedic materials. After that, these adherence interactions led to an upregulation of the biofilm-forming genes. This hypothesis is supported by the studies of global gene expression and proteome analysis of *S. aureus* biofilms. These studies have demonstrated that *S. aureus* in the planktonic mode of growth, especially in the exponential phase, can upregulate the production of adhesins for many host adhesive matrix molecules (e.g. fibrinogen, fibrin, osteopontin, fibronectin, collagen, elastin) [23–25].

There are many biofilm assay systems for the production of in vitro biofilms, including the three-channel flow cell, planar flow cell multichannel, and microdevice flow system [26–28]. In all these systems, the flow of media is followed by the inoculate bacteria in to the chamber, which overall is a time-consuming process requiring at least 1 or 2 days for biofilm mass production. In our flow chamber system, we could achieve a biofilm mass in less than 1 day in salted media, and also in a very short period of time with human plasma media. In addition, the benefit of our system is that it can be used to test different types of orthopaedic materials.

In our current study, we explored the kinetics of *S. aureus* biofilm formation by qRT-PCR analysis of 16S rRNA, which is an indicator of cell viability. To the best of our knowledge, the present study is the first report to describe the use of a qRT-PCR assay for the quantification of *S. aureus* on orthopaedic implants. Our results showed that the living bacteria attached to the orthopaedic material in a time-dependent manner to form an in vitro biofilm in our flow chamber. With this technique, we could detect bacteria at an early time point (3 h) and we also demonstrated a higher sensitivity than SEM and BLI measurement for detecting bacteria loaded onto orthopaedic implants. Our findings correspond to those found in the previous studies of Wada et al. [29] and Bergin et al. [30], who reported that rRNA qRT-PCR is a sensitive and reliable test as a viability indication for bacteria in clinical samples and periprosthetic infections, respectively.

Biofilm formation on implants is a major cause of chronic infection following orthopaedic surgery [9–11]. Thus, there is a great demand for anti-microbial-coated implants that can significantly inhibit biofilm formation. As suspected, here we demonstrated that this problem is exacerbated by strain-specific biofilm phenotypes that must be accounted for to protect against *S. aureus* infections. While anti-adhesin coatings may be effective for strains like SH1000 that directly bind to the surface of metal implants, this approach would likely be ineffective against MSSA strains like UAMS-1 that indirectly bind to the implant via host factors [31]. Moreover, the need for a combined anti-microbial coating approach is highlighted by the highly virulent MRSA strain USA300, which is capable of forming biofilms via host factor-dependent and -independent mechanisms equally well on SS and Ti K-wires [32].

While the primary goal of this study was to develop a rapid in vitro biofilm assay system for the quantitative measurement of *S. aureus* growth and biofilm formation with morphological similarities to that observed in animal models and on retrieved implants from patients with *S. aureus* infection, it is not a substitute for in vivo research [22]. Also, one major limitation of this system is the absence of host immune mechanisms and other critical biological components of the bone microenvironment; hence, follow-up studies to confirm these findings in an appropriate in vivo model are warranted. In addition, in our system, we used only three strains of *S. aureus*, which does not represent the full range of *S. aureus* involved in orthopaedic biofilm infections.

## Conclusion

*S. aureus* is the most common pathogen involved in orthopaedic implant infection and biofilm formation, which is a critical event in the pathogenesis of orthopaedic infection. Here, we demonstrated a novel in vitro model for *S. aureus* biofilm formation with quantitative BLI and SEM outcome measures, and then used this model to demonstrate the presence of strain-specific phenotypes and the model’s potential use to evaluate anti-microbial surfaces.

## Materials and methods

### *S. aureus* strains and growth conditions

Three different *S. aureus* strains were used in this study: 1) SH1000, which has been extensively studied as a robust biofilm-producing strain in static biofilm assays [33], 2) UAMS-1 (and its bioluminescent version Xen40 (Caliper, Alameda, CA, USA)), which is a prototypical methicillin-sensitive *S. aureus* (MSSA) strain isolated from an osteomyelitis patient [34], and 3) USA300 LAC (and its bioluminescent version USA300 LAC::lux), which is the most prevalent community-acquired methicillin-resistant *S. aureus* (MRSA) strain [35]. USA300 LAC::lux was provided by Dr. Tammy Kielian. The strains Xen40 and USA300LAC::lux both possess the bioluminescent *lux*ABCDE operon construct from the bacterial insect pathogen *Photorhabus luminescens*, which naturally produces a blue-green light, but emits luminescent light only when it is alive and metabolically active. All the *S. aureus* strains were stored at − 80 °C in Tryptic Soy Broth (TSB; Sigma Aldrich, Missouri, USA) containing 10% glycerol. For the in vitro biofilm experiments, all the strains were cultured in TSB and grown overnight at 37 °C in a shaking incubator. The bacterial inoculants (2 × 10^8^ cfu/ml) were estimated by measuring the absorbance at 600 nm.

### Orthopaedic implant materials

#### Stainless-steel (SS) pins

Flat (0.5 mm wide by 0.2 mm deep) stainless-steel ribbons (type 304v) were obtained from MicroDyne Technologies (Plainville, CT, USA), and cut to pin lengths of 7 mm for use in the flow chamber assay.

#### Kirschner-wire (K-wire)

Round orthopaedic-grade K-wires in both stainless-steel 316 L (SS316L) and titanium (Ti-6Al-4 V) [21] materials were obtained from Synthes (Monument, CO, USA) with a diameter of 1.25 mm.

All the materials were coated with 20% normal human plasma at 4 °C overnight prior to placement in the flow chamber.

### Flow chamber used for the biofilm formation

A flow chamber that could continuously circulate the bacterial strains of interest at 37 °C was used to evaluate the formation of in vitro biofilms on the stainless-steel flat pins. A one-channel flow chamber with channel dimensions of 2.5 × 7.5 × 2 mm was inserted with a microscope glass cover slide from Leica Biosystems (Richmond, IL, USA) and the stainless-steel flat pins were placed on top. TSB supplemented with 0.5% dextrose and 3% NaCl (TSBGN) and TSB with 10% human plasma (Biological Specialty Corporation, Colmar, PA, USA) (TSB + HP) were used as the biofilm media. The media flow was initiated at a constant rate of 0.2 ml/min (fluid velocity = 0.534 mm s^− 1^) using a Bio-Rad low-pressure pump (Model EP-1 Econo Pump) (Bio-Rad Life Science Research, Hercules, CA, USA). Then, overnight cultures (1:200 dilutions) were inoculated in 300 ml of the biofilm medium (final concentration of bacteria 1 × 10^6^ cfu/ml) and circulated through a cassette containing the stainless-steel pins. After the specified incubation periods, the pins were removed from the flow chamber and analysed to assess their bioluminescence intensity (BLI), morphological appearance by SEM, and for qRT-PCR analysis of the biofilm formation.

### Quantification of the bioluminescence

Biofilm formation on the stainless-steel pins was quantified from the bioluminescent signal arising from the biofilms on the pins. The bioluminescence was imaged with a CCD camera (IVIS® Lumina II, Imaging System, Caliper Life Sciences, Hopkinton, MA, USA) directly after incubation using a 12.5 cm field of view, binning of 4, 1/f stop, 60 s exposure time, and open filters, with automatic correction for the background luminescence. The circular regions of interest (ROIs) were set to 1 × 1 cm to coincide with the size of the stainless-steel pins. The total photon flux over the ROIs was converted to an average radiance (p/s/cm^2^/sr) using Living Image® software (Caliper Life Sciences).

### Scanning electron microscopy (SEM) imaging and analysis

The stainless-steel pins were removed at the set incubation time point in the flow chamber and the biofilm that had formed on them was fixed in 2.5% glutaraldehyde/4.0% paraformaldehyde, then post-fixed in 1.0% osmium tetroxide, before being dehydrated in 100% ethanol, and then placed into a critical point dryer to remove any remaining water and so that the ethanol could be exchanged with CO_2_ gas for complete specimen drying. A Zeiss-Auriga field emission scanning electron microscope was utilized to examine the biofilm/pins and digital images were captured using a Gatan digital system. Quantification of the biofilm on the pins was performed by ROI analysis of a 150x SEM image, which included an entire surface of the pin, and the % surface covered by biofilm determined by ImageJ 1.46r software (Wayne Rasband, NIH, USA), reported as the mean +/− standard error.

### Immunogold-labelling of the fibronectin network

For immunogold-labelling, the biofilm formed from the UAMS-1 ∆*spa* strain, which is a protein A negative strain of *S. aureus*, was used. Immunogold-labelling was done for the SEM analysis to confirm the presence of fibronectin in the biofilm matrix on the K-wires. The wires were fixed for 30 min in 4.0% paraformaldehyde in 0.1 M Millonig’s buffer, then rinsed in phosphate-buffered saline (PBS), blocked using 1.0% normal goat serum in 0.1% bovine serum albumin (BSA) in PBS for 1 h at room temperature, followed by incubation in polyclonal rabbit anti-human fibrinogen (Dako) at 1:200 dilution overnight at 4 °C. After rinsing six times in PBS, the wires were incubated for 2 h at room temperature in a 1:40 dilution of a gold-tagged (30 nm) goat anti-rabbit secondary antibody (Structure Probe, Inc.) diluted in 0.1% BSA/PBS. The wires were rinsed in PBS and post-fixed in 2.0% glutaraldehyde/PBS overnight and then processed for SEM examination. No sputter coating was done on the wires prior to the imaging.

### Kinetics of the in vitro biofilm formation

We determined the kinetics of in vitro biofilm formation by the two *S. aureus* strains by incubating the pins for 0, 3, 6, 9, and 18 h in the flow chamber at 37 °C (*n* = 4). The extent of biofilm formation for each sample was determined from its bioluminescent emission and from SEM photographs of the ROI, which was used to quantify the % surface covered by the biofilm using ImageJ 1.46r software (Wayne Rasband, NIH, USA).

### RNA extraction and quantitative real-time polymerase chain reaction (qRT-PCR) analysis

The bacterial biofilms on the stainless-steel pins were harvested at the desired inoculation time points, washed with TSB media to remove any planktonic bacteria, and stored at − 80 °C overnight before RNA extraction. The total bacteria RNA was extracted from the bacteria using the RNeasy Mini Kit (Qiagen, Inc., Valencia, California) according to the manufacturer’s instructions. DNA contamination was eliminated by means of on-column DNase digestion prior to elution of the total RNA with 30 μl RNase-free water. The amount of recovered RNA was determined spectrophotometrically, and the absence of DNA was verified by PCR. The RNA was converted to cDNA using the iScript cDNA synthesis kit following the manufacturer’s instructions (Bio-Rad Laboratories). After that, quantitative real-time PCR was performed for the *S. aureus* 16S rRNA gene to quantify the bacteria load utilizing the 16S rRNA primers forward 5′-CCAGACTCCTACGGGAGGCAG-3′ and reverse 5′-CGTATTACCGCGGCTGCT-3′ to amplify the 200-bp product. Briefly, the reactions were carried out in a final volume of 10 μl, consisting of 300 nM primer, iQ™ SYBR® Green Super Mix (2X) (Bio-Rad, Hercules, CA, USA), and 1 μl of the cDNA template. The samples were assayed in triplicate in a Rotor-Gene RG3000 system (Corbett Research, Sydney, AU). In order to calculate the 16S rRNA gene copies in a pin sample, we first generated a standard curve with *S. aureus* 16S rRNA purified directly from an overnight culture. The mean of three cycle threshold (Ct) values from each sample was then plotted against this standard curve to extrapolate the number of 16S rRNA genes.

### qRT-PCR analysis of the biofilm-forming genes

To determine the relative expression level of the *icaA, fnbA, spa*, and *alt* genes, which are the important genes that participate in biofilm formation, qRT-PCR analysis of these genes was performed. RNA from the 9-h UAMS-1 biofilm and 6-h USA300 biofilm were reverse transcribed and subsequently analyzed by qRT-PCR as described previously. The sequences of the primers were *gyrB* forward 5′-CCAGGTAAATTAGCCGATTGC-3′, *gyrB* reverse 5′-AAATCGCCTGCGTTCTAGAG-3′, *icaA* forward 5′-AACAGAGGTAAAGCCAACGCACTC-3′, *icaA* reverse 5′-CGATAGTATCTGCATCCAAGCAC-3′, *fnb* forward 5′-ACAGTAACAGAACAACCGTCAAACG-3′, *fnb* reverse 5′-TTGCTGGTTGTGCAGTTTGTG-3′, *spa* forward 5′-TTAGCATCTGCATGGTTTGC-3′, *spa* reverse 5′-AAGAAGACGGCAACGGAGTA-3′, and *alt* forward 5′-TACCGTAACGGCGTAGGTCGT-3′, *alt* reverse 5′-CATAGTCGTGTGTGTGTACGA-3′. The relative expression levels were determined by comparison with the level of *gyrB* expression in the same cDNA preparations.

### Statistical analysis

All the values are expressed herein as the mean +/− standard error (SE). Statistical analysis was performed using StatView for Windows version 5 (SAS Institute Inc., Cary, NC, USA). The Mann–Whitney U test was used to compare the nonparametric values. A *p*-value < 0.05 was considered to be statistically significant.

## Data Availability

All data generated or analysed during this study are included in this published article.

## Abbreviations

TSB + HP: Tryptic Soy Broth with 10% human plasma
TSBGN: Tryptic Soy Broth with dextrose and NaCl
BLI: Bioluminescence intensity
SEM: Scanning electron microscopy
K: Kirchner wire
Ti: Titanium wire
SS: Stainless steel
ROI: Region of interest
